# Risk of venous thromboembolism with janus kinase inhibitors in inflammatory immune diseases: a systematic review and meta-analysis

**DOI:** 10.3389/fphar.2023.1189389

**Published:** 2023-06-07

**Authors:** Juqi Zhang, Wenhui Li, Mingli Gong, Yanlun Gu, Hanxu Zhang, Bingqi Dong, Qi Guo, Xiaocong Pang, Qian Xiang, Xu He, Yimin Cui

**Affiliations:** ^1^ Department of Pharmacy, Peking University First Hospital, Beijing, China; ^2^ Institute of Clinical Pharmacology, Peking University First Hospital, Beijing, China; ^3^ Department of Pharmacy Administration and Clinical Pharmacy, School of Pharmaceutical Sciences, Peking University, Beijing, China; ^4^ Department of Pharmacy, Xu Zhou Medical University, Xuzhou, China; ^5^ Department of General Surgery, Peking University First Hospita, Beijing, China; ^6^ Department of Pharmacy, Peking University Third Hospital, Beijing, China

**Keywords:** janus kinase inhibitor, venous thromboembolism, inflammatory immune diseases, meta-analysis topic: venous thromboembolism, deep vein thrombosis, pulmonary embolism

## Abstract

**Objectives:** This study aimed to evaluate the risk of venous thrombosis (VTE) associated with Janus kinase (JAK) inhibitors in patients diagnosed with immune-mediated inflammatory diseases.

**Methods:** We conducted a comprehensive search of PUBMED, Cochrane, and Embase databases for randomized controlled trials evaluating venous thromboembolic incidence after administering JAK inhibitors in patients with immune-mediated inflammatory diseases. The studies were screened according to the Preferred Reporting Items for Systematic Reviews and Meta-Analyses (PRISMA) guidelines, and a meta-analysis was performed.

**Results:** A total of 16 studies, enrolling 17,242 participants, were included in this review. Four approved doses of JAK inhibitors were administered in the included studies. The meta-analysis revealed no significant difference in the incidence of VTE between patients receiving JAK inhibitors, a placebo, or tumor necrosis factor (TNF) inhibitors (RR 0.72, 95% CI (0.33-1.55); RR 0.94, 95%CI (0.33-2.69)). Subgroup analysis showed a lower risk of VTE with lower doses of JAK inhibitors [RR 0.56, 95%CI (0.36-0.88)]. Compared with the higher dose of tofacitinib, the lower dose was associated with a lower risk of pulmonary embolism [RR 0.37, 95%CI (0.18-0.78)].

**Conclusion:** Our meta-analysis of randomized controlled trials observed a potential increase in the risk of VTE in patients with immune-mediated inflammatory diseases treated with JAK inhibitors compared to placebo or tumor necrosis factor inhibitors, though statistical significance was not attained. Notably, a higher risk of pulmonary embolism was observed with high doses of tofacitinib. Our findings provide valuable insights for physicians when evaluating the use of JAK inhibitors for patients with immune-mediated inflammatory diseases.

**Systematic Review Registration:** https://www.crd.york.ac.uk/prospero/display_record.php?ID=CRD42023382544, identifier CRD42023382544

## Introduction

Immune-mediated inflammatory diseases (IMIDs) are a prevalent and clinically diverse group of chronic inflammatory diseases, with a prevalence of 3%–7% in developed countries (Buckley et al., 2021). IMIDs include chronic inflammatory arthritis (rheumatoid arthritis (RA), spondyloarthritis (SpA) disease spectrum), connective tissue diseases, inflammatory skin diseases (including psoriasis and atopic dermatitis), inflammatory bowel disease (IBD), asthma, and autoimmune neurological diseases such as multiple sclerosis. The disease is characterized by relapsing exacerbations, often accompanied by comorbidities such as cardiovascular disease, cognitive impairment, and skeletal disease. As the disease progresses, there are varying degrees of organ damage, significantly increasing the risk of death. Traditionally, treatment has been based on broad-spectrum immunomodulation, with IMIDs classified according to the clinical type of organ involved. However, traditional therapies, including broad-spectrum immunomodulators, are often associated with severe adverse effects and diminishing efficacy (Mundo et al., 1997; Wang et al., 2018; Pfaller et al., 2020; McInnes and Gravallese, 2021). Cytokine-targeted immunotherapy has transformed the treatment of immune-mediated diseases as the pathogenesis of inflammatory autoimmune diseases has become better understood (Schwartz et al., 2016; Smolen et al., 2020).

Janus kinase (JAK) inhibitors are a new class of targeted synthetic disease-modifying anti-rheumatic drugs (tsDMARDs). As small molecule inhibitors, JAK inhibitors act on the JAK-STAT pathway to block one or more intracellular tyrosine kinases, including JAK1, JAK2, JAK3, and TYK2, mediating various immune regulatory processes by interfering with multiple cytokine signaling pathways (Schwartz et al., 2017).

JAK inhibitors are now commonly utilized in patients who have not responded well to conventional therapies. A second generation of JAK inhibitors has been developed, and currently, nine JAK inhibitors are available worldwide. Their primary indications include rheumatoid arthritis, myelofibrosis, psoriatic arthritis, ulcerative colitis, and graft-versus-host disease.

JAK inhibitors present promising options for managing chronic inflammatory diseases. However, healthcare providers and patients should remain aware of the potential risk of venous thrombosis during treatment. Several clinical trials have established that patients with rheumatoid arthritis receiving JAK inhibitor treatment, particularly those with cardiovascular or venous thromboembolic (VTE) risk factors at baseline, have a significantly higher incidence of thromboembolic events, cancer, and death compared to those treated with anti-TNF agents (Ytterberg et al., 2022; Charles-Schoeman et al., 2023; Curtis et al., 2023). In response to these safety concerns, the FDA issued a boxed warning for JAK inhibitors - including tofacitinib, baricitinib, and upadacitinib - in 2021 (Administration UFaD, 2021). These safety risks apply to all FDA-approved indications for JAK inhibitors, including rheumatoid arthritis, psoriatic arthritis, juvenile idiopathic arthritis, axial spondyloarthritis, ulcerative colitis, atopic dermatitis, and pemphigus (Agency, 2023). When weighing the potential advantages and risks associated with JAK inhibitor treatment, a thorough assessment of individual patient factors, including cardiovascular and VTE risk, is essential.

The mechanism of VTE is multifactorial, and the Virchow triad, which includes damage to the vessel wall, increased blood coagulability, and venous stasis, is a traditional theory linking it (Brotman et al., 2004). IMIDs boost the risk of VTE in the same three ways. Chronic inflammation damages the vessel wall and might impact endothelial functions beyond the physical destruction (Olech and Merrill, 2006; Smeeth et al., 2006). Patients with IMIDs often have varying degrees of pain and localized swelling, which limit movement, compress the affected area, and eventually lead to venous stasis, affecting regional blood flow (Previtali et al., 2011). Studies indicate that individuals suffering from inflammatory immune diseases are significantly more prone to pulmonary embolism (PE) and VTE than the control population (Romero-Díaz et al., 2009; Yusuf et al., 2015; Ketfi et al., 2021; Shaheen and Silverberg, 2021; Bieber et al., 2022). In theory, using JAK inhibitors could increase the risk of deep venous thrombosis (DVT), particularly in patients with preexisting thrombotic risk factors (Molander et al., 2020). The mechanisms leading to this paradoxical phenomenon remain insufficiently explained, and they might be partly linked to thrombogenic operations. The JAK/STAT pathway participates in thrombosis during platelet production or has a role in platelet functions that might relate to maturation processes such as aggregation. (Miyakawa et al., 1996).

In view of the high incidence of PE and VTE in patients with inflammatory immune diseases, and the increasing use of JAK inhibitors, this study aims to investigate the incidence of VTE associated with JAK inhibitors in the target population, based on clinical study data and comparison with two control groups. Through subgroup analysis, the study aims to identify the risk of VTE and PE associated with different types and doses of JAK inhibitors, and to provide guidance and support to clinicians in developing personalized immunotherapy regimens, assessing patients’ risks, and selecting appropriate drugs.

## Methods

### Databases and search strategy

We comprehensively searched human studies through 7 November 2022, without a defined start date. Our search was performed in PubMed, Cochrane, and Embase using the following search string: “tofacitinib or baricitinib or peficitinib or upadacitinib or filgotinib” and “rheumatoid arthritis, psoriatic arthritis, spondyloarthritis, ulcerative colitis, Crohn’s disease, lupus erythematosus, or psoriasis.” Sample search terms are provided in Supplementary Material S1. Two researchers (ZJQ and GML) conducted the initial search.

### Eligibility criteria

Eligible studies were original reports of phase II and phase III randomised clinical trials (RCTs) of JAK inhibitor therapy with a placebo comparator arm. Studies that lacked a double-blind design were omitted. After confirming that the original papers were included in the search, long-term extension (LTE) studies, *post hoc* analysis, and pooled analyses were omitted. Abstracts of conferences, case reports, letters to the editor, review papers, case–control studies and cohort studies were omitted. As of the literature search date of 7 November 2022, all authorized doses of the JAK inhibitors included in the study were considered. The following doses were evaluated: tofacitinib at 5 mg and 10 mg twice daily, baricitinib at 2 mg and 4 mg once daily, upadacitinib at 7.5 mg, 15 mg, 30 mg, and 45 mg once daily, and fingotinib at 100 mg and 200 mg once daily.

### Study selection

Two researchers independently evaluated the titles and abstracts of relevant papers and selected those that met the criteria for inclusion. A third researcher resolved disagreements on the inclusion of a study. Three researchers extracted information from relevant studies and entered it into a collection table. Studies that were later deemed ineligible following a comprehensive assessment of the transcripts were excluded. To verify that there was no duplication, the national clinical trial numbers of the included studies were compared.

### Data extraction

The following details were retrieved from each study: citation information, author list, study design, underlying ailment, study duration, study location, number of patients, inclusion/exclusion criteria, drug doses, patient characteristics, and adverse events (AEs). Deep vein thrombosis (DVT) and pulmonary embolism (PE) were classified as VTE-related occurrences. Full-text articles, additional materials, and appendices were mined for information regarding these events. To confirm that all VTE events were recognised, an extra evaluation of the tabular summary of the original RCT data was undertaken in the ClinicalTrials.gov database. Three separate researchers reviewed all of the data included in the meta-analysis.

### Assessment of bias

Each study undergoing data extraction was assessed for quality using the Cochrane risk-of-bias tool (Supplementary Figure S1).

### Statistical analysis

The Revman5.4 program was used for the analysis. Using the Mantel‒Haenszel random-effects approach for binary data, risk ratio (RR) and 95% confidence intervals were calculated to evaluate the pooled relative risk of VTE with JAK inhibitor therapy *versus* placebo and TNF inhibitor. Subgroup analyses were performed for four JAK inhibitor classes and doses. Forest plots graphically depict the estimates.

## Results

### Study screening

**FIGURE 1:**
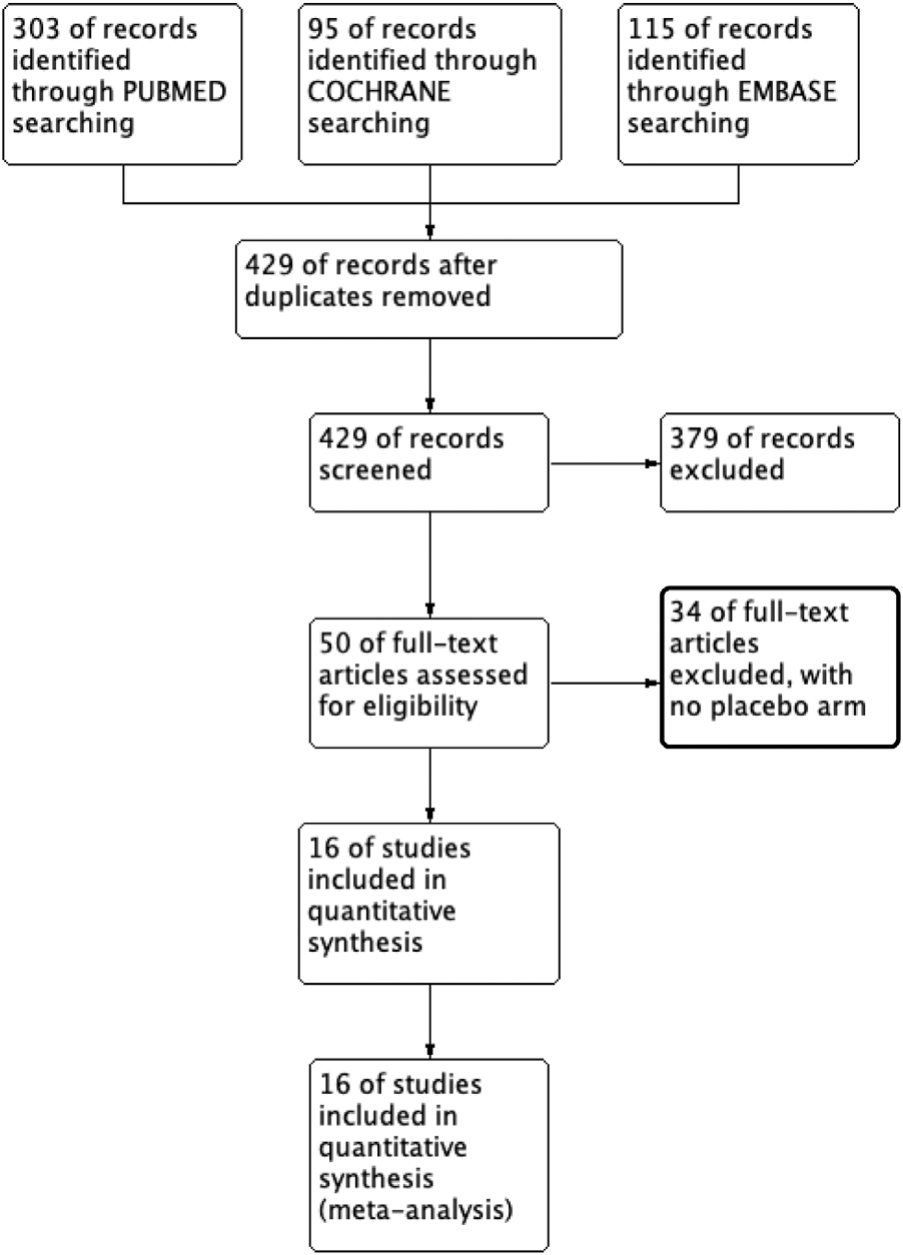
Search results and study selection.

Upon searching the electronic database, a total of 513 articles were identified. After evaluating each article’s title and abstract, 429 were ineligible. A complete read-through was performed from the remaining 50 articles, leading to the exclusion of an additional 34 articles for failure to meet the eligibility criteria. Only 16 articles remained eligible, as presented in Supplementary Material S2. Figure 1 provides a flowchart illustrating the systematic literature review.

### Study characteristics

This meta-analysis includes 16 studies with a total of 17,242 patients, one of which was designated as a phase II/III study. The studies were published between 2013 and 2022, with 10,918 patients receiving JAK inhibitors, 3,119 patients receiving a placebo, and 3,205 patients receiving TNF inhibitors. Seven studies focused on rheumatoid arthritis, five on ulcerative colitis, three on psoriatic arthritis, and one on systemic lupus erythematosus. Supplementary Table S1 presents a detailed summary of all the randomized controlled trials (RCTs) included in this analysis.

### Meta-analysis

**FIGURE 2:**
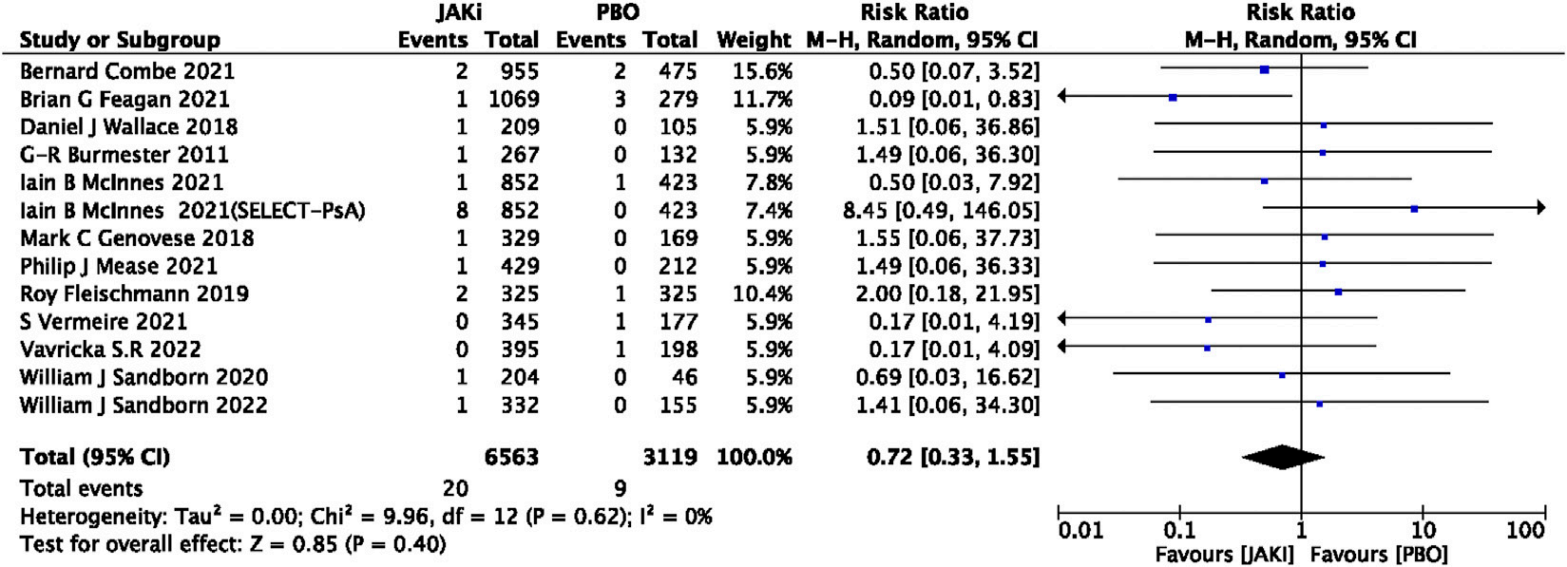
Forest plot of Jaki vs. PBO venous thromboembolism events. JAKi = JAK inhibitors; PBO = placebo; 95% CI = 95% confidence interval; *p* < 0.05 was considered statistically significant.

**FIGURE 3:**
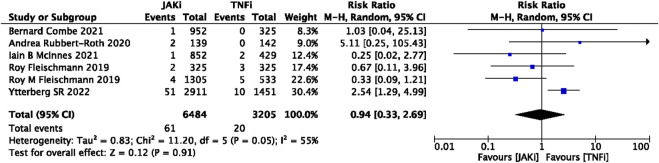
Forest plot of Jaki vs. TNFi venous thromboembolism events. JAKi = JAK inhibitors; TNFi = TNF inhibitors; PBO = placebo; 95% CI = 95% confidence interval; *p* < 0.05 was considered statistically significant.

**FIGURE 4:**
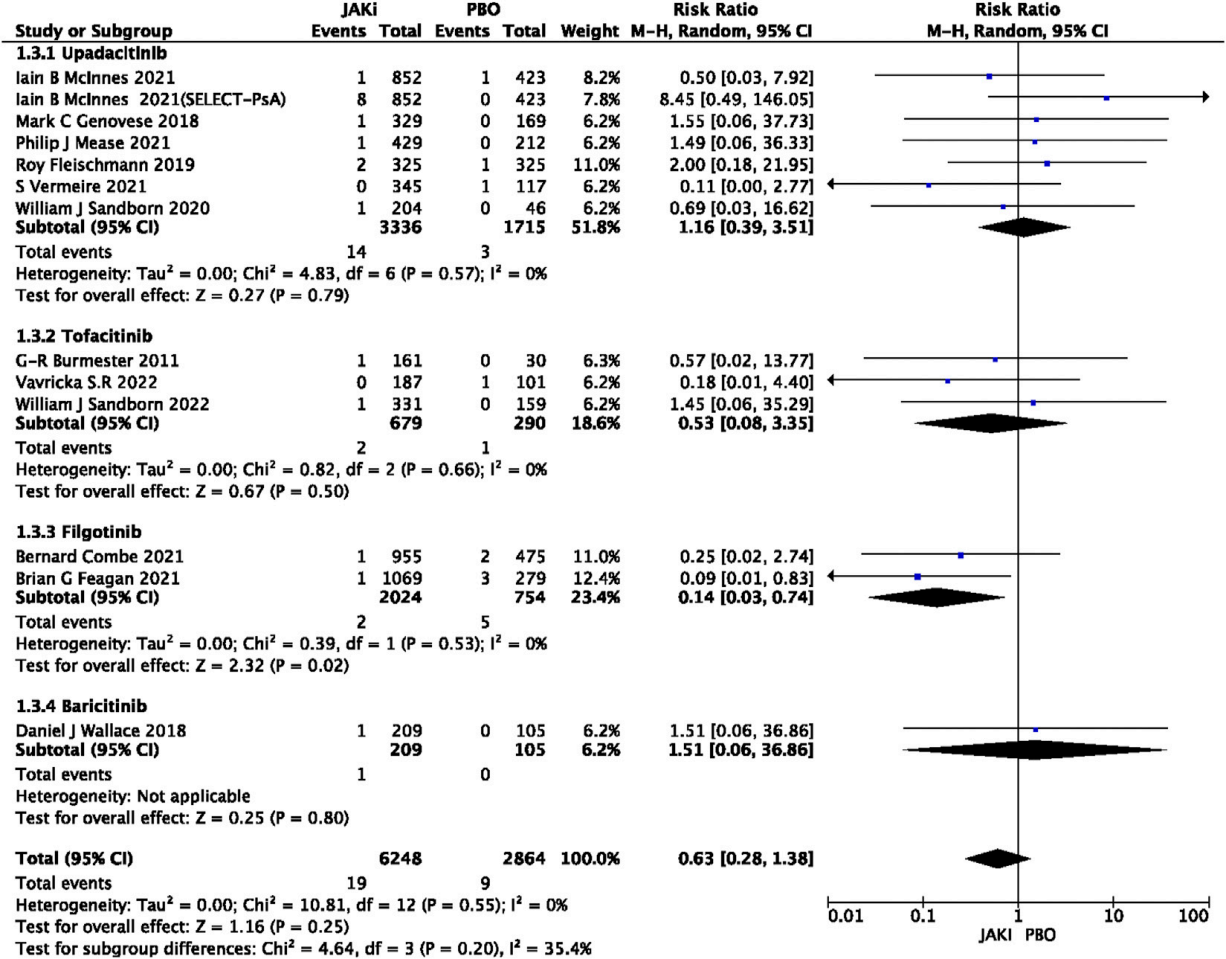
Forest plot of VTE in JAK vs. PBO for drug subgroup analysis.

**FIGURE 5:**
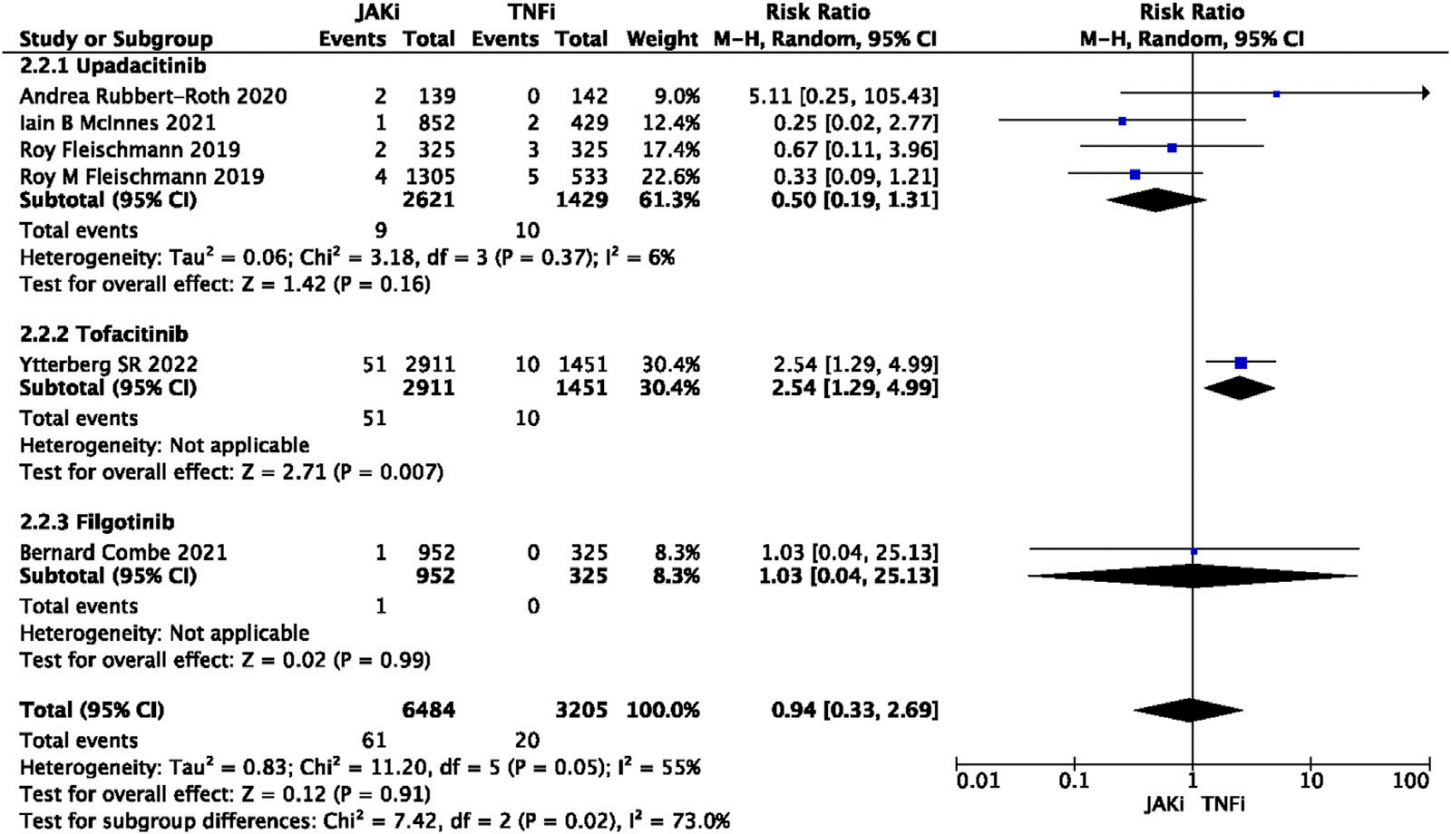
Forest plot of VTE in JAKi vs. TNFi for drug subgroup analysis.

**FIGURE 6:**
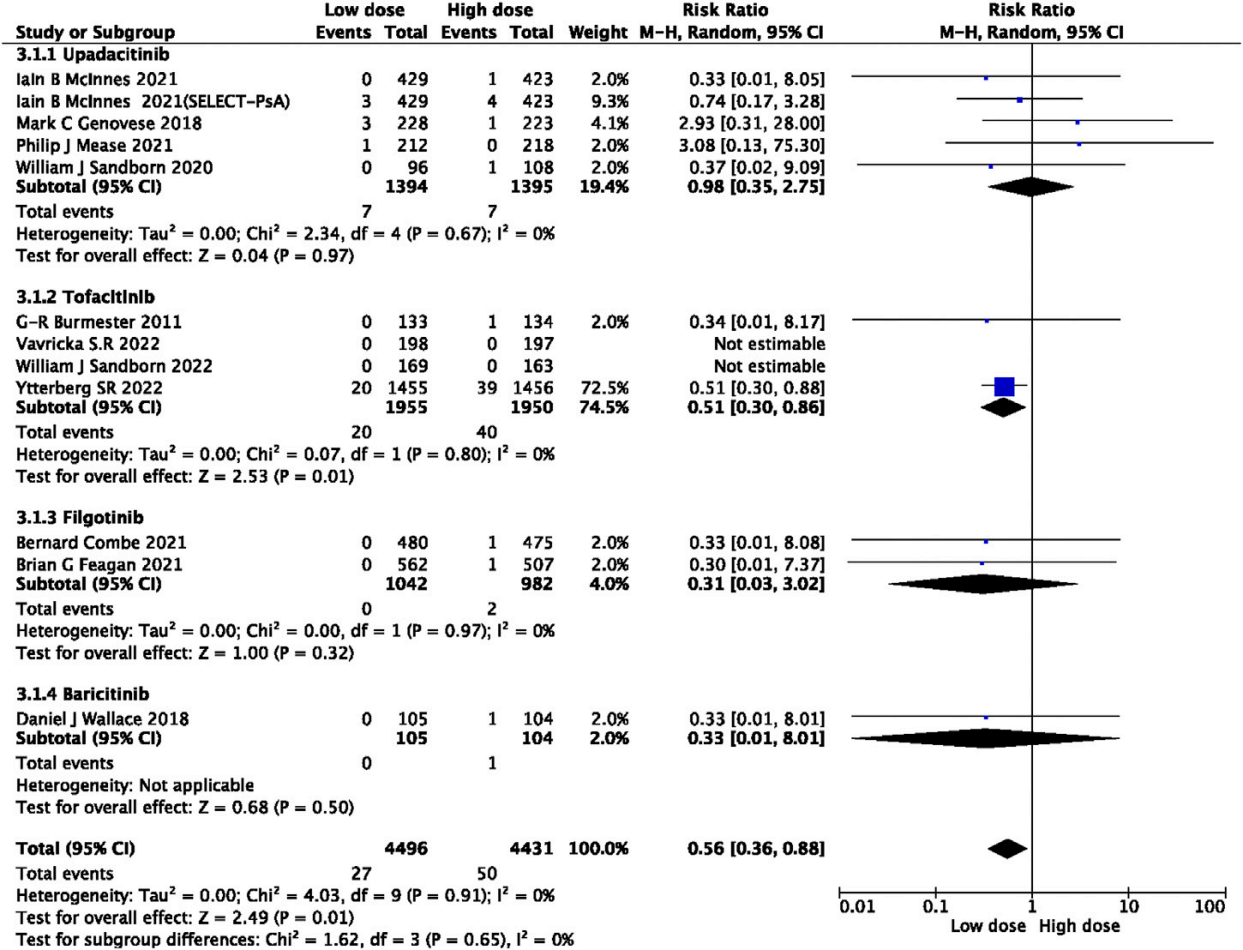
Forest plot of VTE for subgroup analysis of drug doses.

In this study, 79 VTE events were reported in patients treated with JAK inhibitors, while 9 VTE events were reported in patients treated with a placebo, and 20 VTE events were reported in patients treated with TNF inhibitors. The pooled risk ratio (RR) for the JAK inhibitor *versus* placebo group was 0.72 (95% CI 0.33, 1.55) (Figure 2), and the pooled RR for the JAK inhibitor *versus* TNF inhibitor group was 0.94 (95% CI 0.33, 2.69) (Figure 3). Further subgroup analyses were conducted examining the specific types of JAK inhibitors used in the studies. Additional details are presented in Figures 4–6. Notably, the subgroup analysis comparing JAKi with placebo as the control group demonstrated a statistically significant reduction in the risk of VTE in patients treated with filgotinib compared to placebo [RR 0.14 (0.03, 0.74); *p* = 0.02] (Figure 4), while the analysis of the other group showed a higher risk of VTE with tofacitinib compared to TNF inhibitors [RR 2.54(1.29,4.99); *p* = 0.007] (Figure 5). Additionally, further subgroup analyses regarding dose showed that the overall number of VTE events was higher with higher doses of JAK inhibitors, and the risk of VTE was higher with tofacitinib at higher doses compared to lower doses [RR 0.51(0.30,0.86); *p* = 0.01] (Figure 6).

### Risk of bias

In general, this selection of research presented a minimal risk of bias. Thirteen studies (81%) were randomised and double-blind (regarding participants and assessors), while 11 studies (69%) had a low risk of bias overall. Relevant data on the possible bias of individual studies can be found in the Supplementary Figure S1.

## Publication bias

For the Mantel–Haenszel fixed-effect, funnel plot analysis showed no evidence of publication bias in all comparisons (Supplementary Figure S2).

## Discussion

We have acquired safety data on four JAK inhibitors from the most recent randomized controlled trials in various IMIDs, including rheumatoid arthritis, psoriatic arthritis, spondyloarthritis, ulcerative colitis, Crohn’s disease, lupus erythematosus, and psoriasis. To assess the safety of JAK inhibitors, we conducted a systematic review and meta-analysis of randomized controlled trials, comparing JAK inhibitors with TNF inhibitors and placebo controls. With conventional treatments for immune diseases exhibiting adverse side effects, there has been increased attention on biologics and targeted therapies, including JAK inhibitors. Given their promising clinical potential, an array of randomized controlled trials on their safety and efficacy have been published. There are growing concerns about the emerging thrombotic risk of JAK inhibitors, warranting theoretical support. As such, we have expanded our sample size and conducted a meta-analysis based on existing RCTs to mitigate the risk of potential study biases. Our paper provides an updated refinement of clinical trial data relating to JAK inhibitors compared to other meta-analyses (Yates et al., 2021; Chen et al., 2022). Additionally, we provide subgroup analyses of VTE risk with different types of JAK inhibitors, discuss selective differences in mechanisms, and offer recommendations for appropriate VTE risk groups. Our in-depth analysis includes recent randomized controlled trials using approved and marketed JAK inhibitors for autoimmune diseases and analyzes their VTE risk to provide evidence supporting clinical decisions related to adverse events.

This meta-analysis, encompassing 16 RCTs and with a total of 18,448 IMIDs patients, indicates that the incidence of VTE is not significantly higher with JAK inhibitors, as compared to placebo and TNF inhibitors in IMIDs patients (RR 0.71 [0.31, 1.60]; RR 1.28 [0.54, 3.05]). This conclusion is consistent with previous research studies (Yates et al., 2021; Chen et al., 2022).

Several real-world studies have investigated the link between JAKis and VTE risk. One multi-database study evaluated RA patients from 14 real-world data sources in the USA, Europe, and Japan. These patients had received baricitinib treatment compared to TNFi, with an average therapy duration of 9 months. The results indicate an elevated VTE risk in patients who received baricitinib (IRR = 1.51, 95% CI 1.10-2.08) (Salinas et al., 2023). Another *post hoc* analysis of 12,410 patients combined data from the US Corrona registry, the IBM MarketScan study database, and the US FDA Adverse Event Reporting System database. The analysis concluded that RA, PsA, and psoriasis patients taking tofacitinib had a higher VTE rate, and the incidence of arterial thromboembolism (ATE) increased in those patients with cardiovascular or VTE risk factors (Mease et al., 2020). However, RCTs and cohort studies often report more conservative results. A recent large French cohort study of 15,835 patients assessed VTE incidence in RA patients receiving JAKis (upadacitinib, baricitinib) and TNFi. This study found no significant VTE risk (HRw 1.1 [0.7, 1.6], *p* = 0.63) (Hoisnard et al., 2023). However, since VTE events are rare and RCTs have strict inclusion criteria that may impact the analysis, VTE is not usually a primary outcome measure and has no central adjudication. Therefore, the analysis may underestimate the real-world incidence. Since most JAKis have a black box warning, the VTE risk should not be overlooked (Bilal et al., 2021).

In this study, we performed a comprehensive update on relevant RCTs and extracted data from various databases. We divided the control group into a placebo group and one that administered TNF inhibitors. We conducted a sub-group analysis for each JAK inhibitor. Our results showed an increased risk of VTE in the filgotinib group (RR 1.04 [0.03, 0.74]) in the placebo control group, while the tofacitinib group exhibited a lower VTE risk than the TNF inhibitor group in the control group (RR 2.56 [1.30, 5.04]). The other groups did not show significant differences. We further explored the correlation between the findings and the selectivity of JAK targets. The JAK signaling pathway is crucial in activating downstream channels that regulate immune activity. Different JAK and STAT proteins have been associated with various immune mechanisms. Genome-wide association studies (GWAS) have established a relationship between the JAK-STAT pathway and IMIDs, For example, JAK2, Tyk2, STAT1, STAT3, STAT4, and IBD; TyK2, TAT1, SLE; TyK2, STAT4, RA; and TyK2, STAT3, and psoriasis are closely linked to the JAK-STAT pathway (Villarino et al., 2017). The studies we reviewed involved selective JAK1 inhibitors (upadacitinib, filgotinib), a JAK1/2 inhibitor (baricitinib), and a JAK1/3 inhibitor (tofacitinib). Most JAK inhibitors resulted in lower platelet counts, except for baricitinib, which caused transient platelet count increases (Harigai and Honda, 2020). This response may be related to the JAK2- thrombopoietin receptor signal transduction, although the exact role of JAK2 in platelet activation remains unclear (Miyakawa et al., 1995). Parra-Izquierdo et al. reported that JAK1/2 inhibitors decrease platelet adhesion to collagen and inhibit platelet activity *in vitro* (Parra-Izquierdo et al., 2022). Moreover, apart from JAK2, JAK3 is also a crucial regulator of platelet function (Witthuhn et al., 1999), and research has linked the JAK/STAT3 pathway with abnormal platelet biology (Cecati et al., 2013). H E Tibbles et al.'s study showed that JAK3 inhibitors prolong bleeding time and event-free survival in a mouse model of thrombin-induced thromboembolism (Tibbles et al., 2001). However, these findings seem contradictory to the VTE events associated with JAK inhibitors. Previous studies attributed this to off-target effects on other pathways (Yates et al., 2021), which remains an avenue for further investigation.

Our study has demonstrated a higher incidence of VTE in patients receiving a tofacitinib dose of 10 mg twice daily compared to those receiving a dose of 5 mg (RR 0.51 [0.30, 0.86]). Specifically, PE was significantly more frequent in patients receiving the higher dose (RR 0.37 [0.18, 0.78]) (Supplementary Figure S3). These results are in accordance with the recommendations issued by the European Medicines Agency (EMA), based on an open-label clinical trial that focused on safety in patients with RA. The trial revealed that compared to TNF inhibitor therapy, the 5 mg twice daily dose of tofacitinib increased the risk of PE by approximately threefold, while the 10 mg twice daily dose increased the risk by sixfold (EMA, 2020). Following this evidence, the FDA has released a boxed warning against higher tofacitinib doses (10 mg twice daily) concerning their potential for dose-dependent VTE (Administration UFaD). It should be noted, however, that further long-term observational studies are necessary to establish the dose dependency relationship. Based on our findings, we suggest that high-dose JAK inhibitors should be cautiously administered to VTE patients at high risk after a thorough risk-benefit assessment, especially when patients receive tofacitinib and exhibit a risk of PE.

Studies on epidemiology of multiple immune diseases included in this research show that women are at a higher risk of developing such diseases (except for UC), which present greater activity and faster disability progression following menopause (Zandman-Goddard et al., 2007; Lleo et al., 2008; Favalli et al., 2019). Given the gender-specific patterns of disease manifestation, the influence of gender variations in trial recruitment on the outcomes should not be ignored. The RCTs included in the analysis enrolled a greater proportion of females with an average age of 50. However, epidemiological data suggest a higher incidence of VTE in males (Bleker et al., 2014). Therefore, gender differences need to be taken into account when interpreting study results. Additionally, we recognize that the majority of participants in our study were of Caucasian ethnicity, highlighting the need for further research from diverse populations to enable race-specific analyses. Our results also showed that patients with IMIDs with baseline VTE risk factors were more susceptible to thromboembolic events than those without such risk factors. These events were typically associated with more than two risk factors: increasing age, obesity, hypertension, hyperlipidemia, smoking, or a family history of VTE. This particular patient population may be underrepresented in current research, thus underscoring the need for more detailed stratification studies for different risk groups in the future.

The prevalence of VTE in IMIDs is higher compared to the general population (Galloway et al., 2020). However, there are baseline VTE risk variations by disease type. For instance, the relative risk of VTE (PE and/or DVT) in patients with RA is 1.99 (Matta et al., 2009). Studies in Asian populations have shown that patients with IBD are 36 times more prone to developing VTE (Chung et al., 2015; Kim et al., 2022). Similarly, a study based on a UK database showed that the incidence of VTE was 3.7 times higher in patients hospitalized with SLE than in controls (Ramagopalan et al., 2011). Hence, it is advisable for clinicians to diagnose different IMIDs accordingly based on the corresponding baseline VTE risk information. There is some available evidence suggesting that the risk of VTE does not increase in patients with RA treated with TNF inhibitors (Davies et al., 2011). In fact, TNF inhibitors appear to reduce the risk of cardiovascular events in RA patients (Barnabe et al., 2011; Ridker et al., 2017). For patients with high VTE risk, who do not respond to conventional therapies, TNF inhibitors have a relatively safe profile. However, extensive studies are necessary to substantiate this conclusion. In light of the findings from our research, it is essential for clinicians to proactively identify patients with IMIDs and evaluate their risk factors for VTE by establishing a suitable VTE risk assessment system. In addition, physicians should exercise caution while prescribing JAK inhibitors and provide meticulous monitoring and follow-ups to reduce the risk of thromboembolic events and enhance patient prognosis. These measures are critical for optimizing patient care and promoting positive clinical outcomes.

Our study is not without limitations. Firstly, we restricted our study to only RCTs and excluded cohort and pathology-controlled studies. We opted to exclude the long-term studies that did not employ a placebo control group. Our analysis of the RCTs showed that the dosing cycles with JAK inhibitors were significantly longer than those in the control group. Incorporating LTE studies in our analysis could have resulted in a more pronounced disparity in the rates of events per year (PEY) between patients who received JAK inhibitors and the control group. While our stringent screening criteria ensured accuracy, RCTs may not provide real-world outcomes. Moreover, short observation and follow-up periods conflict with the long treatment cycles and duration of immune diseases, emphasizing the need for more long-term studies. Some studies also lacked specific numerical data, which may have impacted the results. Secondly, there are confounding factors when investigating the relationship between JAK inhibitors and VTE. IMIDs are known to increase VTE risk, and studies have reported a higher VTE incidence in RA and SLE patients. (Yafasova et al., 2021). High disease activity also increases VTE risk, which may make it challenging to attribute increased risk solely to JAK inhibitors (Molander et al., 2021). Finally, various studies have limited data on thrombotic risk in patients, and drug combinations like glucocorticoids and NSAIDs remain controversial in their cardiovascular safety for RA patients, which can result in observational study bias. The presence of these confounding factors highlights the need for more comprehensive research in this area (Schjerning et al., 2020; Ocon et al., 2021).

The emerging potential of JAK inhibitors in treating autoimmune diseases warrants the need to implement specific treatment strategies while considering the patient’s unique situation. Efficacy and safety must be weighed comprehensively. In addition, discussions regarding the benefits of JAK inhibitors in combination with other immune therapies, particularly for high-risk populations of VTE, the selection of JAK inhibitors targeting various specificities, and the development of future drugs, are anticipated. This article presents a partial analysis of the safety profile of JAK inhibitors, providing medical professionals with insights into these drugs’ clinical applications.

## Rheumatology key messages

1) The increased risk of VTE with JAK inhibitors is of concern.
2) Evaluation of the risk-benefit ratio for the use of JAK inhibitors in patients with IMID who have high risk factors for VTE.
3) Use high-dose JAK inhibitors with caution in patients with IMIDs at high risk of PE.

## Data Availability

The raw data supporting the conclusion of this article will be made available by the authors, without undue reservation.

## References

[B1] Administration UFaD (2021). FDA requires warnings about increased risk of serious heart-related events, cancer, blood clots, and death for JAK inhibitors that treat certain chronic inflammatory conditions. Available from: https://www.fda.gov/drugs/fda-drug-safety-podcasts/fda-requires-warnings-about-increased-risk-serious-heart-related-events-cancer-blood-clots-and-death .

[B2] Administration UFaD Xeljanz, Xeljanz XR (tofacitinib): Drug Safety Communication - due to an increased risk of blood clots and death with higher dose. Available from: https://www.fda.gov/safety/medical-product-safety-information/xeljanz-xeljanz-xr-tofacitinib-drug-safety-communication-due-increased-risk-blood-clots-and-death .

[B3] Agency E. M. (2023). Janus kinase inhibitors (JAKi). Available from: https://www.ema.europa.eu/en/medicines/human/referrals/janus-kinase-inhibitors-jaki .

[B4] Barnabe C. Martin B-J. Ghali W. A. (2011). Systematic review and meta-analysis: Anti-tumor necrosis factor α therapy and cardiovascular events in rheumatoid arthritis. Arthritis Care Res. Hob. 63 (4), 522–529. 10.1002/acr.20371 20957658

[B5] Bieber T. Feist E. Irvine A. D. Harigai M. Haladyj E. Ball S. (2022). A review of safety outcomes from clinical trials of baricitinib in Rheumatology, dermatology and COVID-19. Adv. Ther. 39 (11), 4910–4960. 10.1007/s12325-022-02281-4 36063279 PMC9443639

[B6] Bilal J. Riaz I. B. Naqvi S. A. A. Bhattacharjee S. Obert M. R. Sadiq M. (2021). Janus kinase inhibitors and risk of venous thromboembolism: A systematic review and meta-analysis. Mayo Clin. Proc. 96 (7), 1861–1873. 10.1016/j.mayocp.2020.12.035 33840525

[B7] Bleker S. M. Coppens M. Middeldorp S. (2014). Sex, thrombosis and inherited thrombophilia. Blood Rev. 28 (3), 123–133. 10.1016/j.blre.2014.03.005 24768093

[B8] Brotman D. J. Deitcher S. R. Lip G. Y. H. Matzdorff A. C. (2004). Virchow's triad revisited. South Med. J. 97 (2), 213–214. 10.1097/01.SMJ.0000105663.01648.25 14982286

[B9] Buckley C. D. Chernajovsky L. Chernajovsky Y. Modis L. K. O'Neill L. A. Brown D. (2021). Immune-mediated inflammation across disease boundaries: Breaking down research silos. Nat. Immunol. 22 (11), 1344–1348. 10.1038/s41590-021-01044-7 34675389

[B10] Cecati M. Sartini D. Pozzi V. Giannubilo S. R. Ferretti F. Stortoni P. (2013). Clues to apoptosis pathway involvement in hemolysis, elevated liver enzyme, and low platelet (HELLP) syndrome and intrauterine growth restriction (IUGR). J. Matern. Fetal Neona 26 (1), 26–31. 10.3109/14767058.2012.722713 22978455

[B11] Charles-Schoeman C. Buch M. H. Dougados M. Bhatt D. L. Giles J. T. Ytterberg S. R. (2023). Risk of major adverse cardiovascular events with tofacitinib versus tumour necrosis factor inhibitors in patients with rheumatoid arthritis with or without a history of atherosclerotic cardiovascular disease: A post hoc analysis from ORAL surveillance. Ann. Rheumatic Dis. 82 (1), 119–129. 10.1136/ard-2022-222259 PMC9811099 36137735

[B12] Chen T-L. Lee L-L. Huang H-K. Chen L-Y. Loh C-H. Chi C-C. (2022). Association of risk of incident venous thromboembolism with atopic dermatitis and treatment with janus kinase inhibitors: A systematic review and meta-analysis. JAMA Dermatol 158 (11), 1254–1261. 10.1001/jamadermatol.2022.3516 36001310 PMC9403856

[B13] Chung W-S. Lin C-L. Hsu W-H. Kao C-H. (2015). Inflammatory bowel disease increases the risks of deep vein thrombosis and pulmonary embolism in the hospitalized patients: A nationwide cohort study. Thromb. Res. 135 (3), 492–496. 10.1016/j.thromres.2014.12.025 25596768

[B14] Curtis J. R. Yamaoka K. Chen Y-H. Bhatt D. L. Gunay L. M. Sugiyama N. (2023). Malignancy risk with tofacitinib versus TNF inhibitors in rheumatoid arthritis: Results from the open-label, randomised controlled ORAL surveillance trial. Ann. Rheumatic Dis. 82 (3), 331–343. 10.1136/ard-2022-222543 PMC9933177 36600185

[B15] Davies R. Galloway J. B. Watson K. D. Lunt M. Symmons D. P. M. Hyrich K. L. (2011). Venous thrombotic events are not increased in patients with rheumatoid arthritis treated with anti-TNF therapy: Results from the British society for Rheumatology biologics register. Ann. Rheumatic Dis. 70 (10), 1831–1834. 10.1136/ard.2011.153536 PMC3168333 21784722

[B16] EMA (2020). EMA fifinal recommendations on VTE risk with tofacitinib RheumNow. Available from: https://rheumnow.com/content/ema-fifinal-recommendations-vte-risk-tofacitinib .

[B17] Favalli E. G. Biggioggero M. Crotti C. Becciolini A. Raimondo M. G. Meroni P. L. (2019). Sex and management of rheumatoid arthritis. Clin. Rev. Allergy Immunol. 56 (3), 333–345. 10.1007/s12016-018-8672-5 29372537

[B18] Galloway J. Barrett K. Irving P. Khavandi K. Nijher M. Nicholson R. (2020). Risk of venous thromboembolism in immune-mediated inflammatory diseases: A UK matched cohort study. RMD Open 6 (3), e001392. 10.1136/rmdopen-2020-001392 32994362 PMC7547545

[B19] Harigai M. Honda S. (2020). Selectivity of janus kinase inhibitors in rheumatoid arthritis and other immune-mediated inflammatory diseases: Is expectation the root of all headache? Drugs 80 (12), 1183–1201. 10.1007/s40265-020-01349-1 32681420 PMC7395017

[B20] Hoisnard L. Pina Vegas L. Dray-Spira R. Weill A. Zureik M. Sbidian E. (2023). Risk of major adverse cardiovascular and venous thromboembolism events in patients with rheumatoid arthritis exposed to JAK inhibitors versus adalimumab: A nationwide cohort study. Ann. Rheumatic Dis. 82 (2), 182–188. 10.1136/ard-2022-222824 36198438

[B21] Ketfi C. Boutigny A. Mohamedi N. Bouajil S. Magnan B. Amah G. (2021). Risk of venous thromboembolism in rheumatoid arthritis. Jt. Bone Spine 88 (3), 105122. 10.1016/j.jbspin.2020.105122 33346109

[B22] Kim S. Y. Cho Y. S. Kim H-S. Lee J. K. Kim H. M. Park H. J. (2022). Venous thromboembolism risk in asian patients with inflammatory bowel disease: A population-based nationwide inception cohort study. Gut Liver 16 (4), 555–566. 10.5009/gnl210190 34789583 PMC9289840

[B23] Lleo A. Battezzati P. M. Selmi C. Gershwin M. E. Podda M. (2008). Is autoimmunity a matter of sex? Autoimmun. Rev. 7 (8), 626–630. 10.1016/j.autrev.2008.06.009 18603021

[B24] Matta F. Singala R. Yaekoub A. Y. Najjar R. Stein P. D. (2009). Risk of venous thromboembolism with rheumatoid arthritis. Thromb. Haemost. 101 (1), 134–138. 10.1160/th08-08-0551 19132199

[B25] McInnes I. B. Gravallese E. M. (2021). Immune-mediated inflammatory disease therapeutics: Past, present and future. Nat. Rev. Immunol. 21 (10), 680–686. 10.1038/s41577-021-00603-1 34518662 PMC8436867

[B26] Mease P. Charles-Schoeman C. Cohen S. Fallon L. Woolcott J. Yun H. (2020). Incidence of venous and arterial thromboembolic events reported in the tofacitinib rheumatoid arthritis, psoriasis and psoriatic arthritis development programmes and from real-world data. Ann. Rheumatic Dis. 79 (11), 1400–1413. 10.1136/annrheumdis-2019-216761 PMC7569391 32759265

[B27] Miyakawa Y. Oda A. Druker B. J. Kato T. Miyazaki H. Handa M. (1995). Recombinant thrombopoietin induces rapid protein tyrosine phosphorylation of Janus kinase 2 and Shc in human blood platelets. Blood 86 (1), 23–27. 10.1182/blood.v86.1.23.bloodjournal86123 7795229

[B28] Miyakawa Y. Oda A. Druker B. J. Miyazaki H. Handa M. Ohashi H. (1996). Thrombopoietin induces tyrosine phosphorylation of Stat3 and Stat5 in human blood platelets. Blood 87 (2), 439–446. 10.1182/blood.v87.2.439.bloodjournal872439 8555464

[B29] Molander V. Bower H. Askling J. (2020). OP0034 does the risk of venous thromboembolism vary with disease activity in rheumatoid arthritis? Ann. Rheumatic Dis. 79 (1), 23.2–4. 10.1136/annrheumdis-2020-eular.353 33032998

[B30] Molander V. Bower H. Frisell T. Askling J. (2021). Risk of venous thromboembolism in rheumatoid arthritis, and its association with disease activity: A nationwide cohort study from Sweden. Ann. Rheumatic Dis. 80 (2), 169–175. 10.1136/annrheumdis-2020-218419 33032998

[B31] Mundo A. Pedone V. Lamanna G. Cervini C. (1997). Sulfasalazine: Side effects and duration of therapy in patients with rheumatoid arthritis. Clin. Ter. 148 (1-2), 7–13. 9377840

[B32] Ocon A. J. Reed G. Pappas D. A. Curtis J. R. Kremer J. M. (2021). Short-term dose and duration-dependent glucocorticoid risk for cardiovascular events in glucocorticoid-naive patients with rheumatoid arthritis. Ann. Rheumatic Dis. 80 (12), 1522–1529. 10.1136/annrheumdis-2021-220577 34215644

[B33] Olech E. Merrill J. T. (2006). The prevalence and clinical significance of antiphospholipid antibodies in rheumatoid arthritis. Curr. Rheumatol. Rep. 8 (2), 100–108. 10.1007/s11926-006-0049-8 16569368

[B34] Parra-Izquierdo I. Melrose A. R. Pang J. Lakshmanan H. H. S. Reitsma S. E. Vavilapalli S. H. (2022). Janus kinase inhibitors ruxolitinib and baricitinib impair glycoprotein-VI mediated platelet function. Platelets 33 (3), 404–415. 10.1080/09537104.2021.1934665 34097573 PMC8648864

[B35] Pfaller B. Pupco A. Leibson T. Aletaha D. Ito S. (2020). A critical review of the reproductive safety of Leflunomide. Clin. Rheumatol. 39 (2), 607–612. 10.1007/s10067-019-04819-4 31758422

[B36] Previtali E. Bucciarelli P. Passamonti S. M. Martinelli I. (2011). Risk factors for venous and arterial thrombosis. Blood Transfus. 9 (2), 120–138. 10.2450/2010.0066-10 21084000 PMC3096855

[B37] Ramagopalan S. V. Wotton C. J. Handel A. E. Yeates D. Goldacre M. J. (2011). Risk of venous thromboembolism in people admitted to hospital with selected immune-mediated diseases: Record-linkage study. BMC Med. 9, 1. 10.1186/1741-7015-9-1 21219637 PMC3025873

[B38] Ridker P. M. Everett B. M. Thuren T. MacFadyen J. G. Chang W. H. Ballantyne C. (2017). Antiinflammatory therapy with canakinumab for atherosclerotic disease. N. Engl. J. Med. 377 (12), 1119–1131. 10.1056/nejmoa1707914 28845751

[B39] Romero-Díaz J. García-Sosa I. Sánchez-Guerrero J. (2009). Thrombosis in systemic lupus erythematosus and other autoimmune diseases of recent onset. J. Rheumatol. 36 (1), 68–75. 10.3899/jrheum.071244 19012362

[B40] Salinas C. A. Louder A. Polinski J. Zhang T. C. Bower H. Phillips S. (2023). Evaluation of VTE, mace, and serious infections among patients with RA treated with baricitinib compared to TNFi: A multi-database study of patients in routine care using disease registries and claims databases. Rheumatol. Ther. 10 (1), 201–223. 10.1007/s40744-022-00505-1 36371760 PMC9660195

[B41] Schjerning A-M. McGettigan P. Gislason G. (2020). Cardiovascular effects and safety of (non-aspirin) NSAIDs. Nat. Rev. Cardiol. 17 (9), 574–584. 10.1038/s41569-020-0366-z 32322101

[B42] Schwartz D. M. Bonelli M. Gadina M. O'Shea J. J. (2016). Type I/II cytokines, JAKs, and new strategies for treating autoimmune diseases. Nat. Rev. Rheumatol. 12 (1), 25–36. 10.1038/nrrheum.2015.167 26633291 PMC4688091

[B43] Schwartz D. M. Kanno Y. Villarino A. Ward M. Gadina M. O'Shea J. J. (2017). JAK inhibition as a therapeutic strategy for immune and inflammatory diseases. Nat. Rev. Drug Discov. 16 (12), 843–862. 10.1038/nrd.2017.201 29104284

[B44] Shaheen M. S. Silverberg J. I. (2021). Association of inflammatory skin diseases with venous thromboembolism in US adults. Arch. Dermatol Res. 313 (4), 281–289. 10.1007/s00403-020-02099-6 32642810

[B45] Smeeth L. Cook C. Thomas S. Hall A. J. Hubbard R. Vallance P. (2006). Risk of deep vein thrombosis and pulmonary embolism after acute infection in a community setting. Lancet (London, Engl. 367 (9516), 1075–1079. 10.1016/S0140-6736(06)68474-2 16581406

[B46] Smolen J. S. Landewé R. B. M. Bijlsma J. W. J. Burmester G. R. Dougados M. Kerschbaumer A. (2020). EULAR recommendations for the management of rheumatoid arthritis with synthetic and biological disease-modifying antirheumatic drugs: 2019 update. Ann. Rheumatic Dis. 79 (6), 685–699. 10.1136/annrheumdis-2019-216655 31969328

[B47] Tibbles H. E. Vassilev A. Wendorf H. Schonhoff D. Zhu D. Lorenz D. (2001). Role of a JAK3-dependent biochemical signaling pathway in platelet activation and aggregation. J. Biol. Chem. 276 (21), 17815–17822. 10.1074/jbc.M011405200 11278899

[B48] Villarino A. V. Kanno Y. O'Shea J. J. (2017). Mechanisms and consequences of Jak-STAT signaling in the immune system. Nat. Immunol. 18 (4), 374–384. 10.1038/ni.3691 28323260 PMC11565648

[B49] Wang W. Zhou H. Liu L. (2018). Side effects of methotrexate therapy for rheumatoid arthritis: A systematic review. Eur. J. Med. Chem. 158, 502–516. 10.1016/j.ejmech.2018.09.027 30243154

[B50] Witthuhn B. A. Williams M. D. Kerawalla H. Uckun F. M. (1999). Differential substrate recognition capabilities of Janus family protein tyrosine kinases within the interleukin 2 receptor (IL2R) system: Jak3 as a potential molecular target for treatment of leukemias with a hyperactive Jak-Stat signaling machinery. Leuk. Lymphoma 32 (3-4), 289–297. 10.3109/10428199909167389 10037026

[B51] Yafasova A. Fosbøl E. L. Schou M. Baslund B. Faurschou M. Docherty K. F. (2021). Long-term cardiovascular outcomes in systemic lupus erythematosus. J. Am. Coll. Cardiol. 77 (14), 1717–1727. 10.1016/j.jacc.2021.02.029 33832598

[B52] Yates M. Mootoo A. Adas M. Bechman K. Rampes S. Patel V. (2021). Venous thromboembolism risk with JAK inhibitors: A meta-analysis. Arthritis and Rheumatology (Hoboken, NJ) 73 (5), 779–788. 10.1002/art.41580 33174384

[B53] Ytterberg S. R. Bhatt D. L. Mikuls T. R. Koch G. G. Fleischmann R. Rivas J. L. (2022). Cardiovascular and cancer risk with tofacitinib in rheumatoid arthritis. N. Engl. J. Med. 386 (4), 316–326. 10.1056/NEJMoa2109927 35081280

[B54] Yusuf H. R. Hooper W. C. Grosse S. D. Parker C. S. Boulet S. L. Ortel T. L. (2015). Risk of venous thromboembolism occurrence among adults with selected autoimmune diseases: A study among a U.S. Cohort of commercial insurance enrollees. Thromb. Res. 135 (1), 50–57. 10.1016/j.thromres.2014.10.012 25456001 PMC4480419

[B55] Zandman-Goddard G. Peeva E. Shoenfeld Y. (2007). Gender and autoimmunity. Autoimmun. Rev. 6 (6), 366–372. 10.1016/j.autrev.2006.10.001 17537382

