# Supplementary figures and images for: Risk of venous thromboembolism with janus kinase inhibitors in inflammatory immune diseases: a systematic review and meta-analysis
Source: Front Pharmacol. 2023 Jun 7;14:1189389. doi: 10.3389/fphar.2023.1189389 (PMC10282754; doi:10.3389/fphar.2023.1189389)

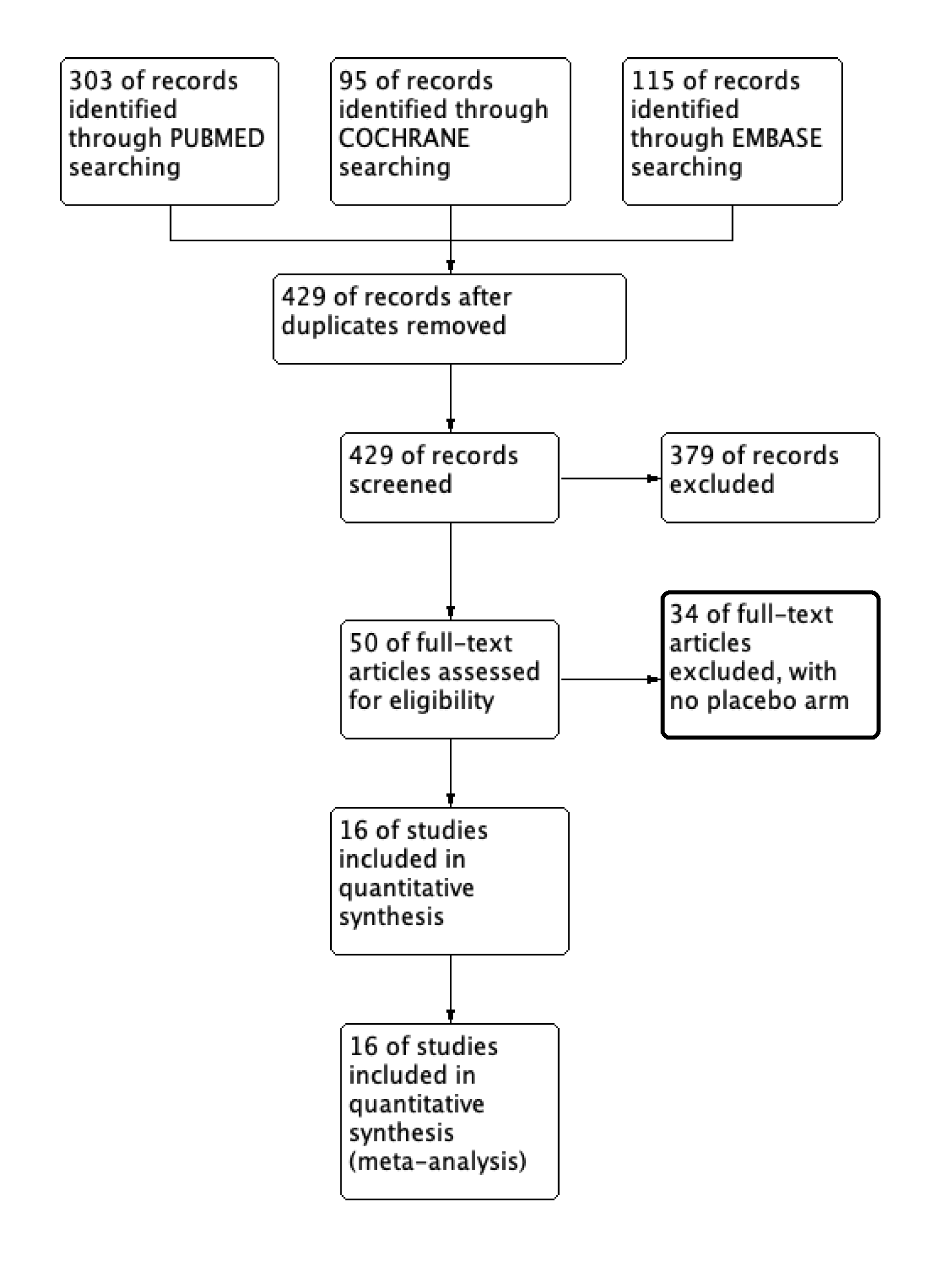

Supplement: Supplementary file 1 [file DataSheet1.ZIP › Figure/Figure1.Flow diagram.tiff]

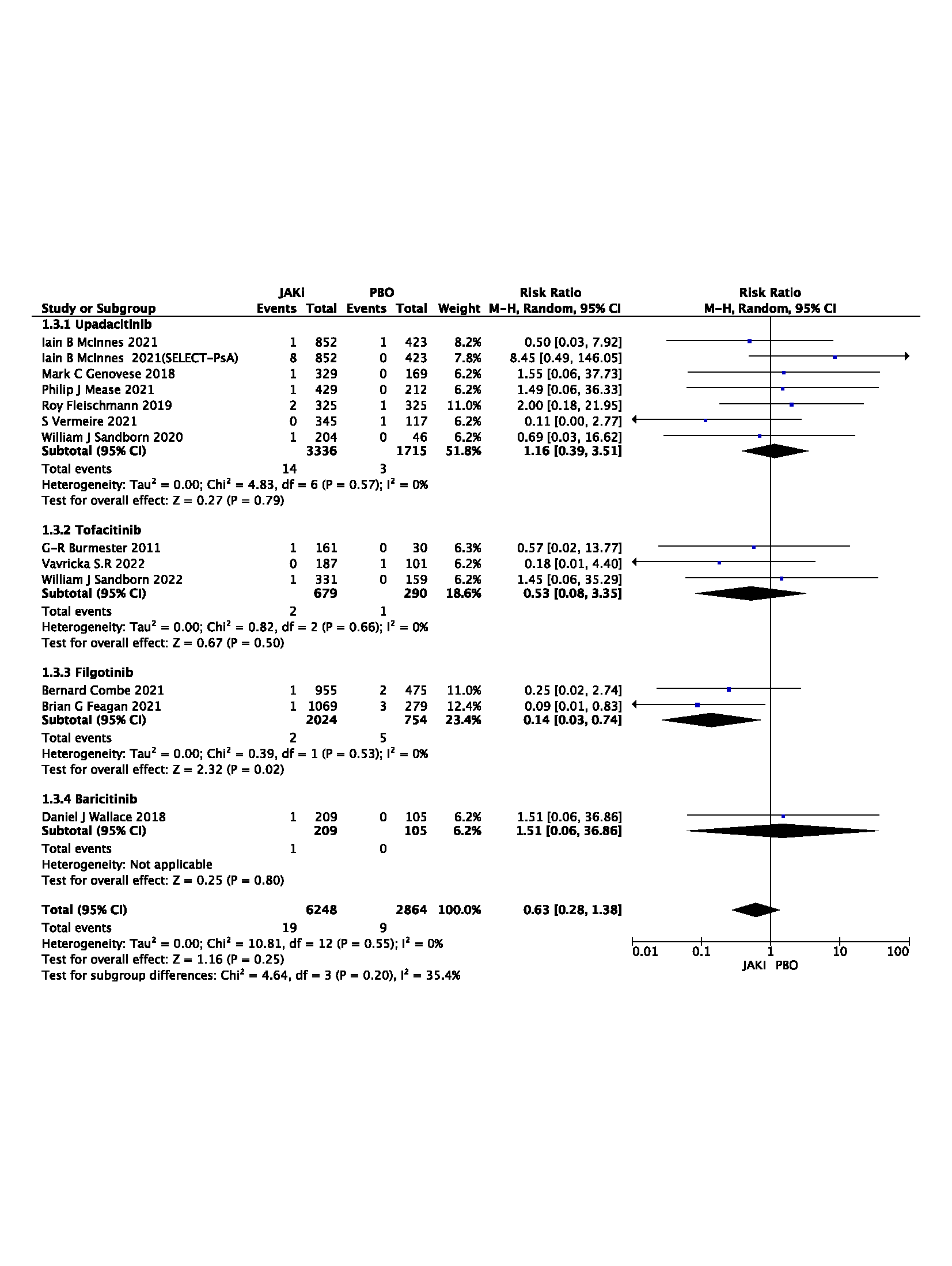

Supplement: Supplementary file 1 [file DataSheet1.ZIP › Figure/Figure4.Drug Subgroup.JAKi VS PBO.tiff]

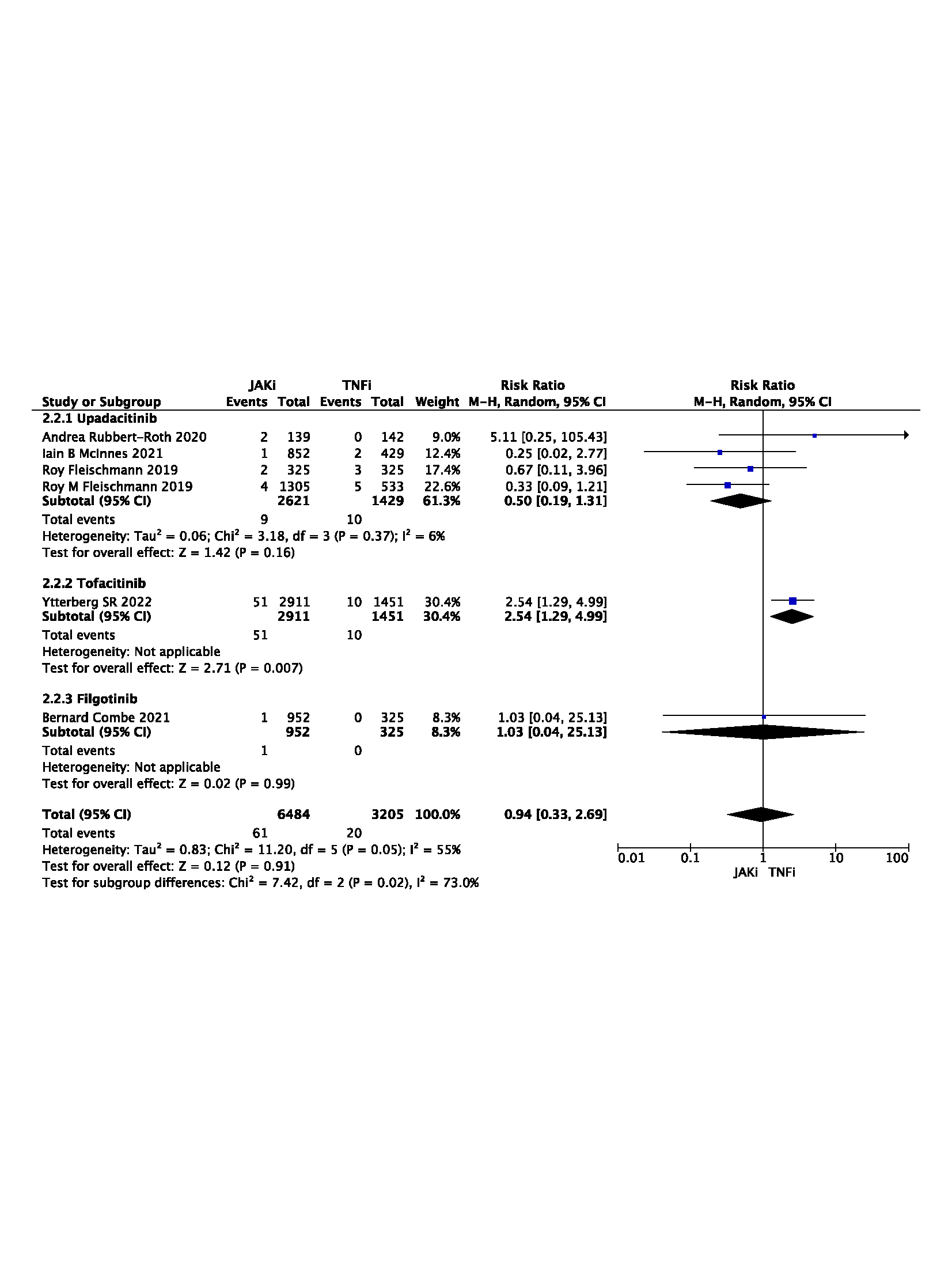

Supplement: Supplementary file 1 [file DataSheet1.ZIP › Figure/Figure5.Drug Subgroup.JAKi VS TNFi.tiff]

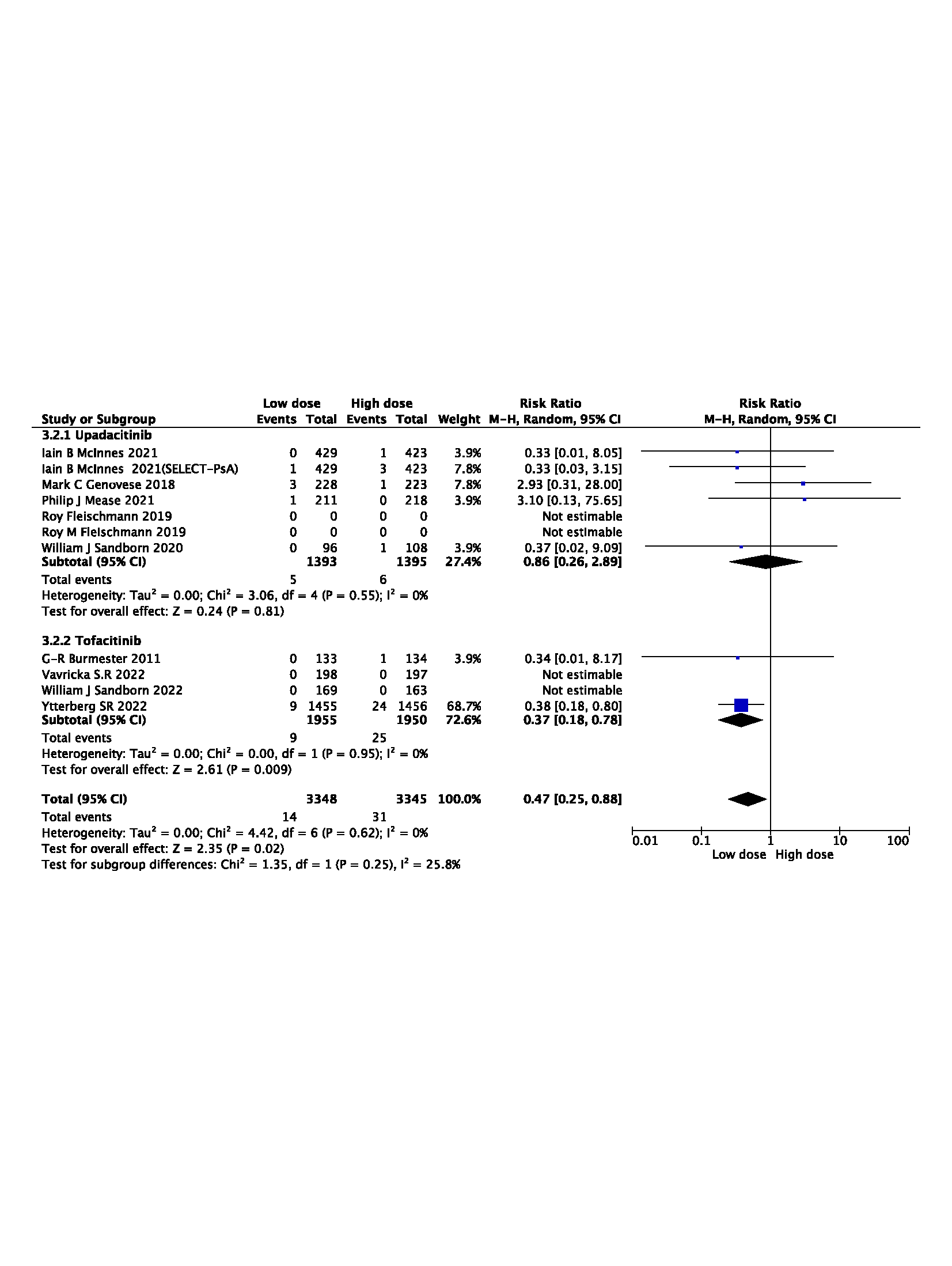

Supplement: Supplementary file 1 [file DataSheet1.ZIP › Figure/Figure S3.PE Forest plot.tiff]

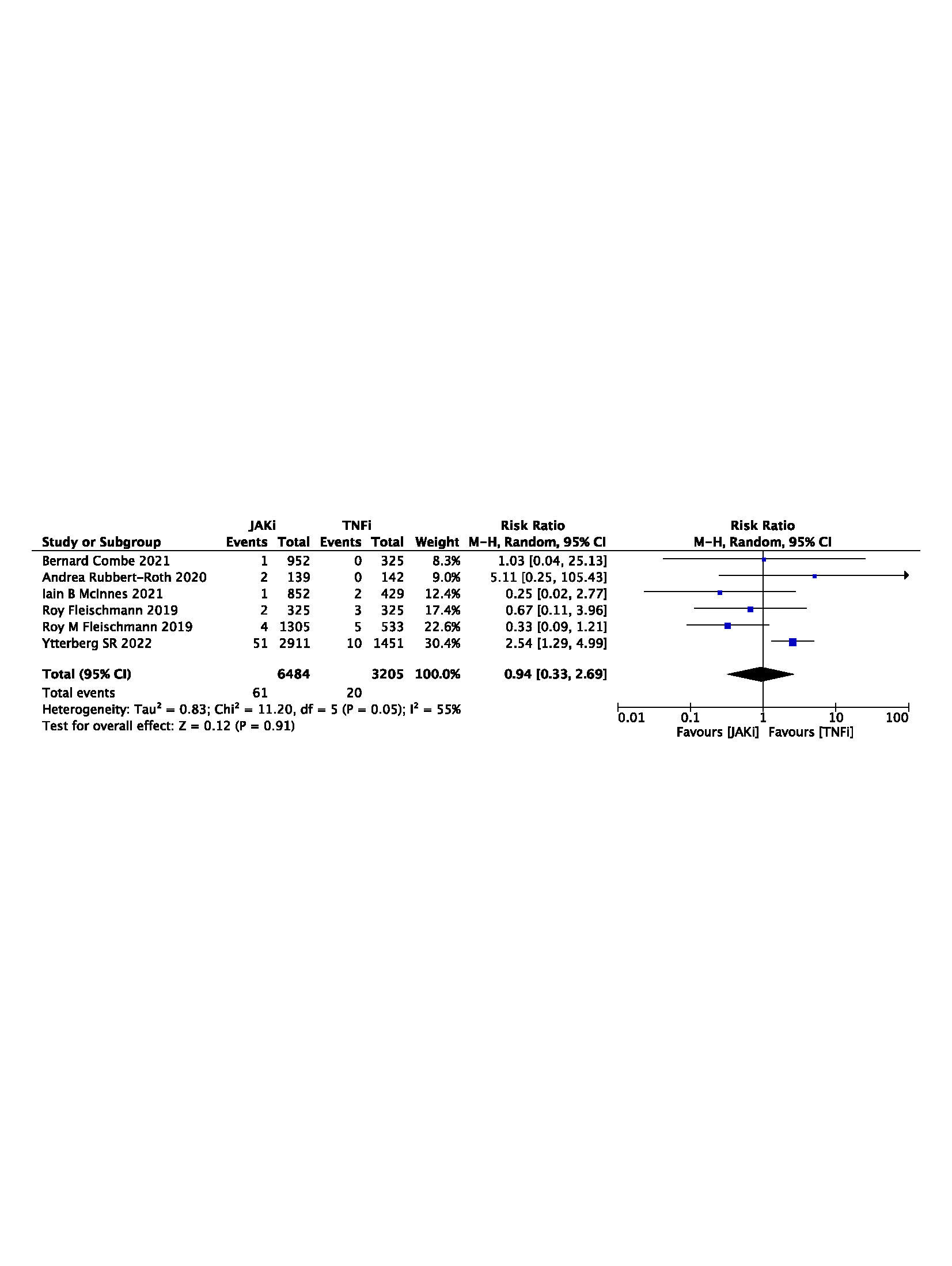

Supplement: Supplementary file 1 [file DataSheet1.ZIP › Figure/Figure3.JAKi VS TNFi-Forest plot.tiff]

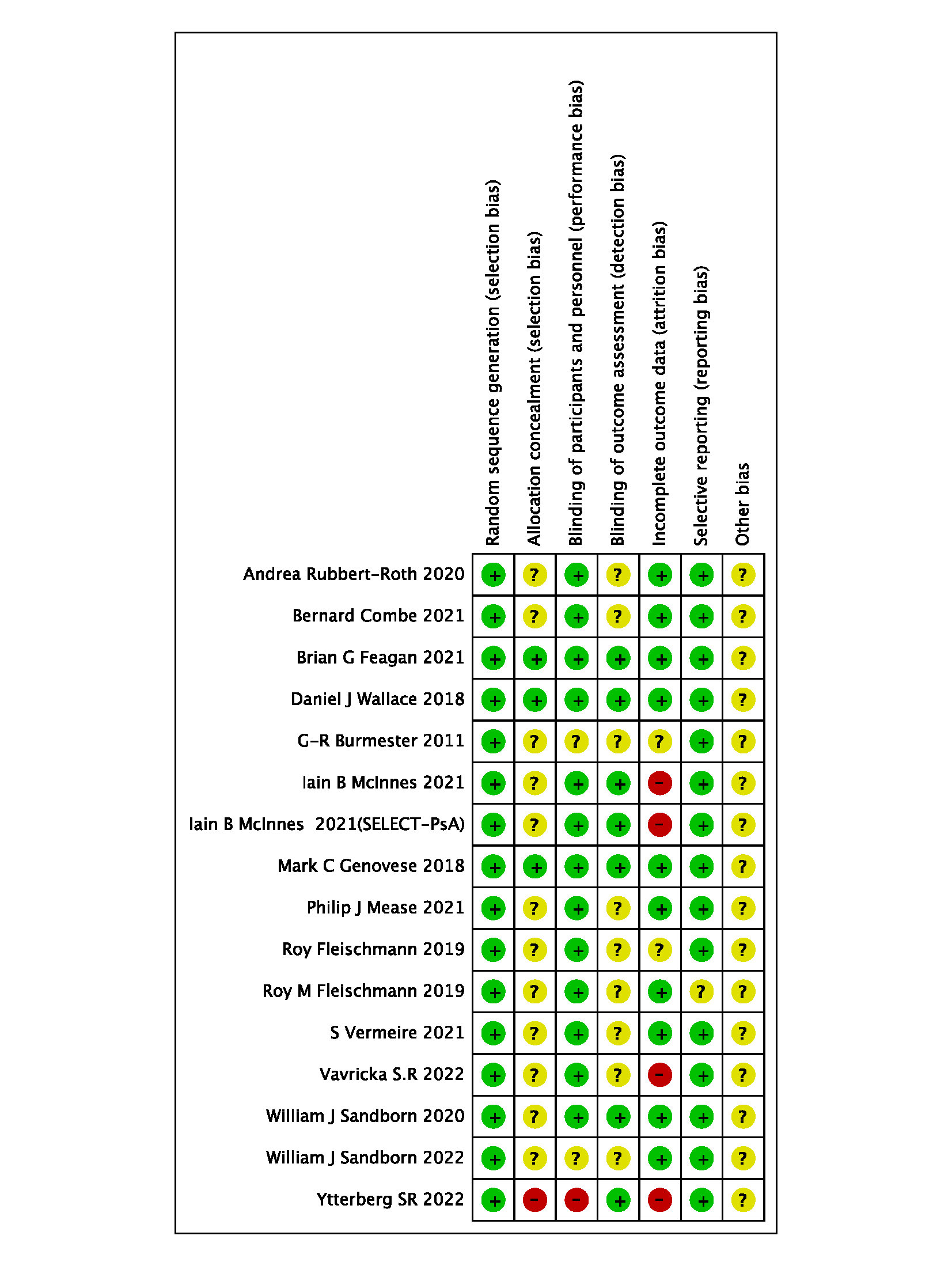

Supplement: Supplementary file 1 [file DataSheet1.ZIP › Figure/Figure S1 Quality assessment.tiff]

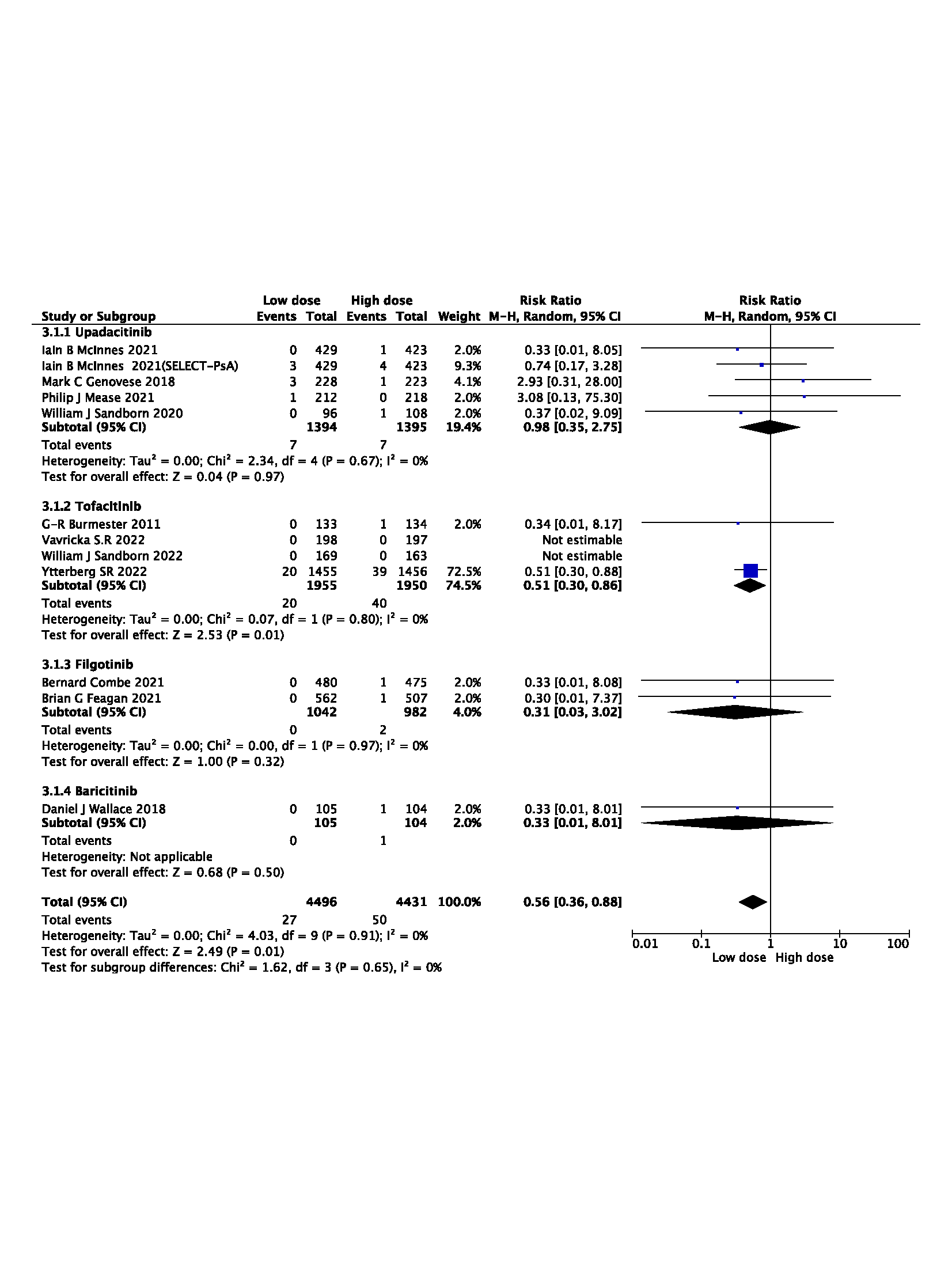

Supplement: Supplementary file 1 [file DataSheet1.ZIP › Figure/Figure6.Dose Subgroup.tiff]

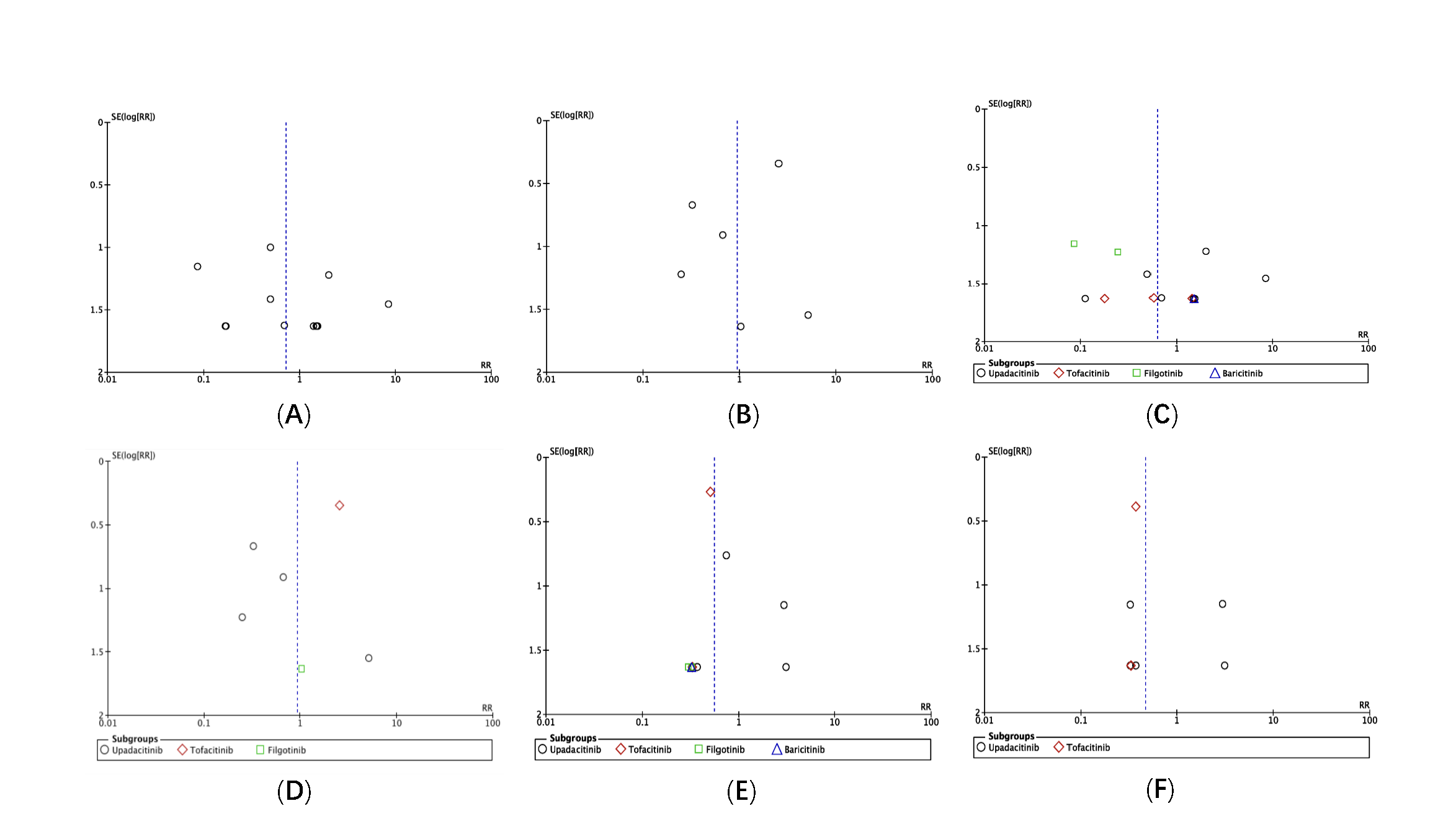

Supplement: Supplementary file 1 [file DataSheet1.ZIP › Figure/Figure S2.Funnel plots.tiff]

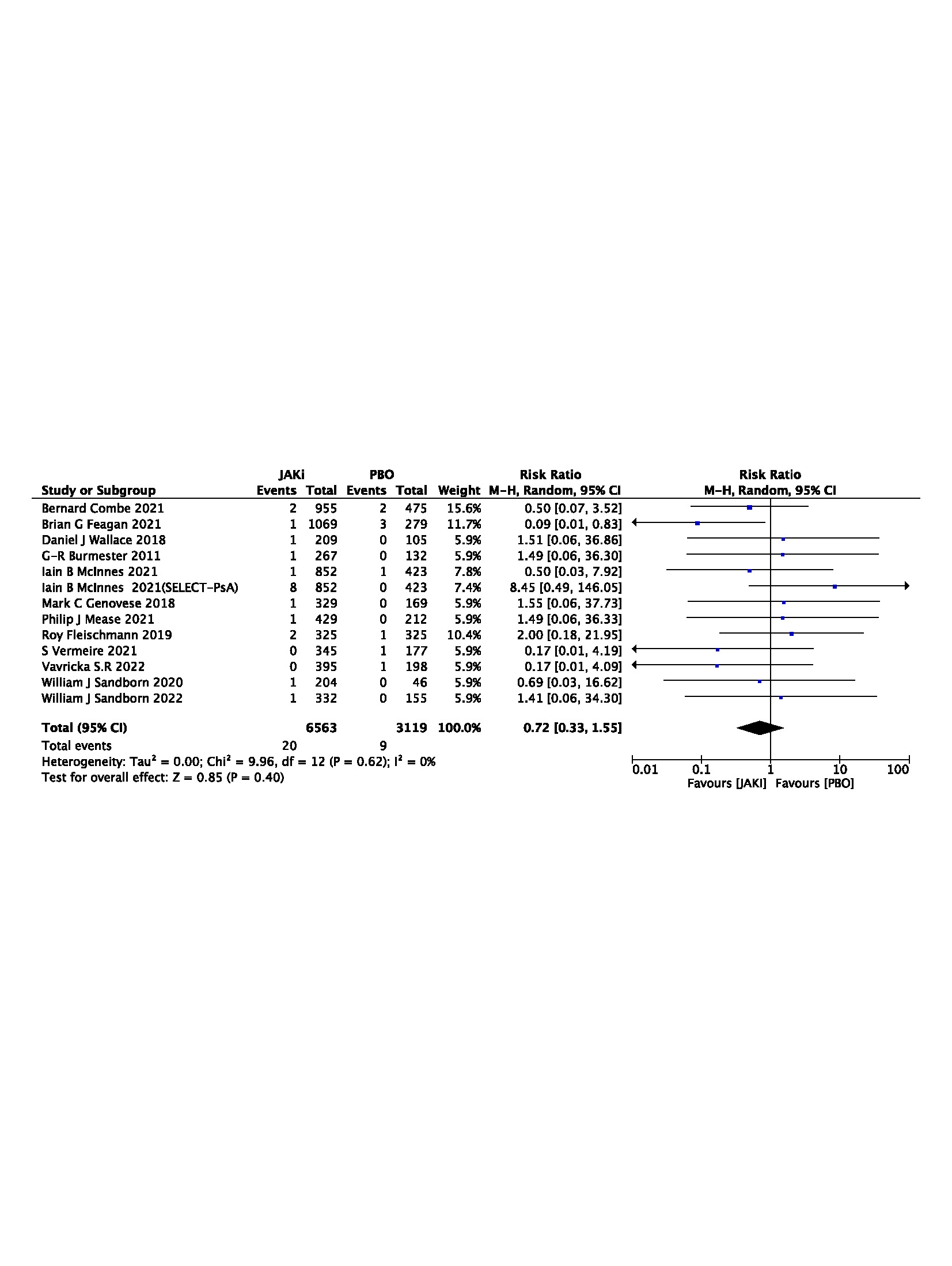

Supplement: Supplementary file 1 [file DataSheet1.ZIP › Figure/Figure2.JAKi VS PBO-Forest plot.tiff]
